# *Candida albicans* cell wall integrity transcription factors regulate polymicrobial biofilm formation with *Streptococcus gordonii*

**DOI:** 10.7717/peerj.7870

**Published:** 2019-10-11

**Authors:** Jennifer Chinnici, Lisa Yerke, Charlene Tsou, Sujay Busarajan, Ryan Mancuso, Nishanth D. Sadhak, Jaewon Kim, Abhiram Maddi

**Affiliations:** Departments of Periodontics & Endodontics and Oral Biology, School of Dental Medicine, State University of New York at Buffalo, Buffalo, NY, United States of America

**Keywords:** Interkingdom interactions, Polymicrobial biofilms, Phenotypic switch, *Streptococcus gordonii*, Ampicillin resistance, *Candida albicans*, Polymicrobial biofilms, Antibiotic tolerance

## Abstract

Polymicrobial biofilms play important roles in oral and systemic infections. The oral plaque bacterium *Streptococcus gordonii* is known to attach to the hyphal cell wall of the fungus *Candida albicans* to form corn-cob like structures in biofilms. However, the role of *C. albicans* in formation of polymicrobial biofilms is not completely understood. The objective of this study was to determine the role of *C. albicans* transcription factors in regulation of polymicrobial biofilms and antibiotic tolerance of *S. gordonii*. The proteins secreted by *C. albicans* and *S. gordonii* in mixed planktonic cultures were determined using mass spectrometry. Antibiotic tolerance of *S. gordonii* to ampicillin and erythromycin was determined in mixed cultures and mixed biofilms with *C. albicans*. Additionally, biofilm formation of *S. gordonii* with *C. albicans* knock-out mutants of 45 transcription factors that affect cell wall integrity, filamentous growth and biofilm formation was determined. Furthermore, these mutants were also screened for antibiotic tolerance in mixed biofilms with *S. gordonii*. Analysis of secreted proteomes resulted in the identification of proteins being secreted exclusively in mixed cultures. Antibiotic testing showed that *S. gordonii* had significantly increased survival in mixed planktonic cultures with antibiotics as compared to single cultures. *C. albicans* mutants of transcription factors Sfl2, Brg1, Leu3, Cas5, Cta4, Tec1, Tup1, Rim101 and Efg1 were significantly affected in mixed biofilm formation. Also mixed biofilms of *S. gordonii* with mutants of *C. albicans* transcription factors, Tec1 and Sfl2, had significantly reduced antibiotic tolerance as compared to control cultures. Our data indicates that *C. albicans* may have an important role in mixed biofilm formation as well as antibiotic tolerance of *S. gordonii* in polymicrobial biofilms. *C. albicans* may play a facilitating role than being just an innocent bystander in oral biofilms and infections.

## Introduction

*Candida albicans* can be found as a commensal in the majority of healthy humans in the oral, vaginal, and gastrointestinal mucosae (Kim & Sudbery, 2011). However, *C. albicans* is also the most common fungal pathogen in humans. It turns opportunistic and causes candidiasis in immunocompromised patients, denture wearers and the elderly (Muzyka & Epifanio, 2013). It is found in over 90% of human fungal infections and is a major etiologic factor in hospital-acquired infections (Azie et al., 2012). With an increasing number of individuals immunocompromised from organ transplantation, chemotherapy, HIV infection, and medical device implantation, infections caused by *C. albicans* are becoming an increasing cause of concern. Candidemia may occur if the fungus is able to disseminate into the bloodstream causing symptoms similar to bacterial septicemia with extremely high mortality rates (Pfaller & Diekema, 2007).

Fungal infections occurring in the mouth, such as oral candidiasis, can become a nidus for disseminated infection (Odds, 1987). *C. albicans* is unique in that it is pleiomorphic and is able to grow in multiple morphological forms. It can exist as yeast (unicellular), hyphae, pseudohyphae and chlamydiospores. The transition from yeast to hyphae is stimulated by multiple factors, including the presence of serum, neutral pH, high CO_2_, N-acetylglucosamine, 37 °C, and when within biofilms (Kim & Sudbery, 2011). The ability of *C. albicans* to phenotypically switch from yeast to hyphal form is associated with virulence, even though both forms are found in infections (Calderone & Fonzi, 2001). *Candida* infections involve formation of a biofilm, which is a structured microbial community adherent to a surface and embedded within a matrix of extracellular polymeric substance. Biofilms in the oral cavity are found on the surface of teeth and dentures. Typically, the dental plaque biofilm is polymicrobial in nature and has *Candida albicans* in close association with several bacteria including *Streptococci,* which are the pioneer bacteria for dental plaque (Jenkinson, Lala & Shepherd, 1990; Ranjan & Dongari-Bagtzoglou, 2018; Xu, Jenkinson & Dongari-Bagtzoglou, 2014).

Streptococcal species most frequently found in the oral cavity, with the exception of *Streptococcus mutans*, the etiologic agent of dental caries, are generally considered beneficial and non-pathogenic (Xu, Jenkinson & Dongari-Bagtzoglou, 2014; Kuramitsu et al., 2007). These commensal bacteria can colonize multiple sites in the oral cavity, including keratinized and non-keratinized oral mucosa, the tongue, periodontal pockets, and teeth. A subgroup of *Streptococcus sp.,* named the mitis group, includes: *Streptococcus gordonii, Streptococcus oralis, Streptococcus mitis, Streptococcus parasanguinis,* and *Streptococcus sanguinis*. The mitis group comprises over 60% of the identifiable oral microbiota (Syed & Loesche, 1978) and dominates the composition of the buccal mucosa in healthy individuals (Diaz et al., 2012). The mitis group is a research focus because of their ability to initiate polymicrobial biofilms in the oral cavity (Whitmore & Lamont, 2011).

*C. albicans* and *S. gordonii* are known to colonize the same habitats within the oral cavity. *Streptococcus sp.* has been verified to form corn-cob like structures around *C. albicans* in dental plaque (Zijnge et al., 2010). Their interactions may therefore be important to commensal colonization in health as well as in pathogenicity. There are several examples of interkingdom interactions between these two microorganisms. *C. albicans* secretes a quorum-sensing molecule, farnesol, that represses hyphae formation at high concentrations (Hornby et al., 2001). However, when *C. albicans* and *S. gordonii* are cultured together, hyphae formation is induced overriding the effect of farnesol (Bamford et al., 2009). Also *S. gordonii,* as with other *Streptococci*, produces lactic acid that may serve as an energy source for *C. albicans*, suggesting another synergistic interkingdom interaction (Jenkinson, Lala & Shepherd, 1990). Additionally, *S. gordonii* was found to have a higher affinity for *C. albicans* compared to other *mitis* bacteria (Holmes, Gopal & Jenkinson, 1995). Bacterial cell wall polypeptides SspA and SspB were identified as adhesion molecules in this interaction (Holmes, McNAB & Jenkinson, 1996). *C. albicans* hyphal wall proteins Als3 and Hwp1 have been identified as receptors for these bacterial polypeptides (Nobbs, Vickerman & Jenkinson, 2010). Attachment between these organisms may enable a variety of cell–cell interactions to occur.

Past studies have shown that *C. albicans* and *S. gordonii* grown together in mixed biofilms have a greater biomass than when cultured in a monospecies biofilm (Xu, Jenkinson & Dongari-Bagtzoglou, 2014; Bamford et al., 2009; Dutton et al., 2014). It has been shown that their interactions induce changes in gene expression that lead to differences in growth, virulence, and drug susceptibility (Dutton et al., 2015). Additionally, a more recent study has shown that polymicrobial biofilms of *C. albicans* and *S. gordonii* have increased resistance to antibiotics (Montelongo-Jauregui et al., 2016). In this study, our primary objective was to understand the functions of *C. albicans* in polymicrobial biofilm formation with *S. gordonii* and antibiotic resistance. In order to better understand the proteomic interactions between these microorganisms we determined secreted proteins unique to planktonic co-cultures of *C. albicans* and *S. gordonii*. We further aimed to demonstrate differences in biofilm formation and anitbiotic tolerance in single species versus mixed biofilms. Additionally, we performed screening of several mutants of *C. albicans* transcription factors that affect cell wall integrity to study regulation of polymicrobial biofilm formation and antibiotic tolerance.

## Materials and Methods

### Strains and culturing conditions

*C. albicans* wild type strain SC5314 and transcription factor knock-out mutant strains (Homann plates) were obtained from the fungal genetics stock center (FGSC). The genetic background and creation of the transcription factor knock-out mutants has been described previously (Homann et al., 2009). The *C. albicans* wild type strain SC5314 and *C. albicans* transcription factor mutant strains were cultured from frozen stock in liquid Yeast Nitrogen Base (YNB) medium (Amresco , OH) with 2% Glucose (Amresco, OH) as a carbon source supplemented with a complete amino acid supplement mixture (CSM) (MP Biomedicals, OH) and grown overnight at 30 °C with shaking at 225 rpm. The *Streptococcus gordonii* wild type strain Challis CH1 was cultured from frozen stock in liquid Tryptic Soy Broth plus 0.3% Yeast Extract (TSBY) medium (Difco, BD Diagnostics, MD) and grown statically overnight at 37 °C in a candle jar. Solid media was prepared by adding 1.5% agar (Alfa Aesar, MA). Both *C. albicans* and *S. gordonii* were also cultured from frozen stock in a mixture of 50% YNB and 50% TSBY in their respective growth conditions where indicated. *C. albicans* cell concentration was monitored with a hemocytometer and *S. gordonii* concentration was monitored by measuring the optical density at 600nm (OD_600_).

### Effect of pH on *C. albicans* Growth

An overnight culture of wild type *C. albicans* in YNB was inoculated from frozen stock. The overnight was used to inoculate 20 ml of five different mixtures of YNB and TSBY: 100% YNB, 75% YNB/25% TSBY, 50% YNB/50% TSBY, 25% YNB/75% TSBY, and 100% TSBY. The pH of each of these mixtures was measured prior to inoculation. The cultures were grown at 30 °C with shaking at 225 rpm. Cell concentrations were taken at 0, 2, 4, and 6 h. A fresh overnight culture of *C. albicans* was used to inoculate five, 20 ml volumes of YNB that had each been adjusted to the pH of one of the YNB/TSBY mixtures used previously. Cultures were grown at 30 °C with shaking and cell concentrations were taken at 0, 2, 4, and 6 h. These cultures served as controls to make sure that alterations in the growth of *C. albicans* were due to the change in media rather than just a change in pH.

### Secreted proteome analysis of co-cultures of *C. albicans* and *S. gordonii*

**Figure 1:**
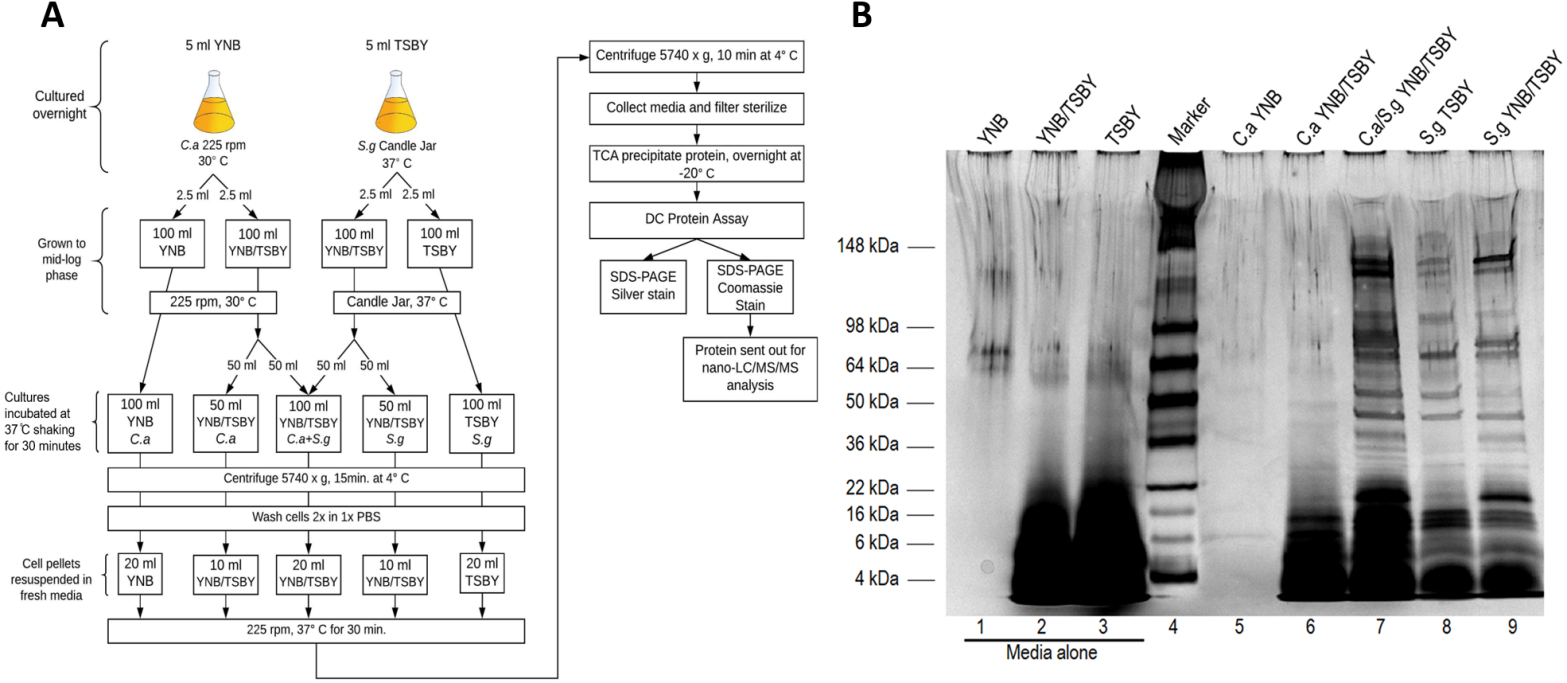
*C. albicans* and *S. gordonii* secreted proteomes in planktonic cultures. (A) Schematic of culture conditions and proteomic analysis of secreted proteins. (B) Silver-stained 4–15% gradient SDS-PAGE gel of TCA precipitated secreted protein from single and co-cultures of 30 minutes only. *C. albicans* was grown in yeast nitrogen base (YNB) and a mixture of yeast nitrogen base and tryptic soy broth with yeast extract (YNB/TSBY) media. *C. albicans and S. gordonii* co-culture was grown in YNB/TSBY. *S. gordonii* was grown in TSBY and YNB/TSBY media. Lanes 1, 2, and 3 contain TCA precipitated proteins from media alone. Proteins 22 kDa and smaller are likely from the TSBY due to the undefined nature of the media. There is no protein from the YNB alone (lane 1) as it is a completely synthetic media with no added protein. Image taken with Gel Doc XR+ and viewed with Image Lab Software (Bio-Rad, USA).

Portions (2.5 ml) of a wild type *C. albicans* overnight culture grown in YNB at 30 °C with shaking were used to inoculate 100 ml of YNB and 100 ml of YNB/TSBY (50/50). Portions (2.5 ml) of a *S. gordonii* overnight culture grown in TSBY statically at 37 °C in a candle jar were used to inoculate 100 ml of TSBY and 100 ml of YNB/TSBY. The two *C. albicans* cultures were grown at 30 °C with shaking and the two *S. gordonii* cultures were grown statically at 37 °C in a candle jar until they reached mid-log phase (approximately 5 × 10^7^ cells/ml and OD_600_ = 0.6 respectively) for a total of four different cultures. Upon reaching desired cell concentration, 50 ml of the *C. albicans* culture grown in YNB/TSBY (50/50) was combined with 50 ml of the *S. gordonii* culture that was grown in the same type of media for a total volume of 100 ml in a new container to make a fifth culture group. The cells from all five groups were harvested by centrifugation (5,470×g, 15 min). The cell pellets were washed twice with cold 1× PBS. The cell pellets from the cultures with a 100 ml volume prior to centrifugation (*C. albicans* in YNB, *S. gordonii* in TSBY, and the YNB/TSBY combined co-culture) were re-suspended a fresh 20ml volume of their respective original medias. The cell pellets from the cultures with a 50 ml volume prior to centrifugation (the remainders of the single cultures of *C. albicans* and *S. gordonii* grown in YNB/TSBY left over from the creation of the co-culture group) were re-suspended in a fresh 10 ml volume of YNB/TSBY. All five cultures were then allowed to grow at 37 °C with shaking for 30 min. After the incubation period, the cells were removed from the media by centrifugation (5,470× g, 10 min.) followed by filtration using 0.2 µm syringe filters (Corning, NY). The proteins in the media were collected for analysis by TCA precipitation using a final concentration of 12.5% TCA (Amresco, OH) and 50% acetone (Fisher Scientific, ON). Proteins were allowed to precipitate for 24 h at −20 °C. In addition to the spent culture media samples, TCA precipitations of each fresh media (YNB, TSBY, or YNB/TSBY) were included as controls to see what protein was coming from the media alone. The precipitated proteins were collected by centrifugation (10,510×g, 10 min.) and washed twice with cold 100% acetone. All samples were re-suspended in a small volume of 1× PBS after allowing the acetone to evaporate completely in a flow hood and subjected to a DC Protein Assay (Bio-Rad, CA) to determine protein concentration. The protein samples were then subjected to SDS-PAGE. 9 µg of protein of each sample were loaded onto a 4 to 15% Mini-Protean TGX gel (Bio-Rad, CA) with the exceptions of the YNB media only control and the *C. albicans* sample that was grown in YNB. For these samples, as much as possible was loaded since there is no protein coming from the YNB itself and *C. albicans* secretes very little protein when grown in YNB. The proteins were visualized using a silver stain kit (Bio-Rad, CA). An additional gel was run for the preparation of samples for mass spectrometric (MS) analysis. 9 µg of each sample were loaded onto a SDS-PAGE gel and subjected to a brief electrophoresis step of 5 min so that the dye front only traveled a short distance into the gel. The gel was then stained with Coomassie Brilliant Blue (Amresco, OH). The protein bands from the co-culture and the single cultures of *C. albicans* and *S. gordonii* (all grown in YNB/TSBY) were cut out of the gel after de-staining and sent for nano-LC/MS/MS analysis at the Fred Hutchinson Cancer Research Center (FHCRC), Seattle, WA. Samples from two repeats of the experiment were sent for MS analysis. Please see Fig. 1A for a flow chart of methods.

### Assessment of ampicillin tolerance in planktonic cultures

**Figure 2:**
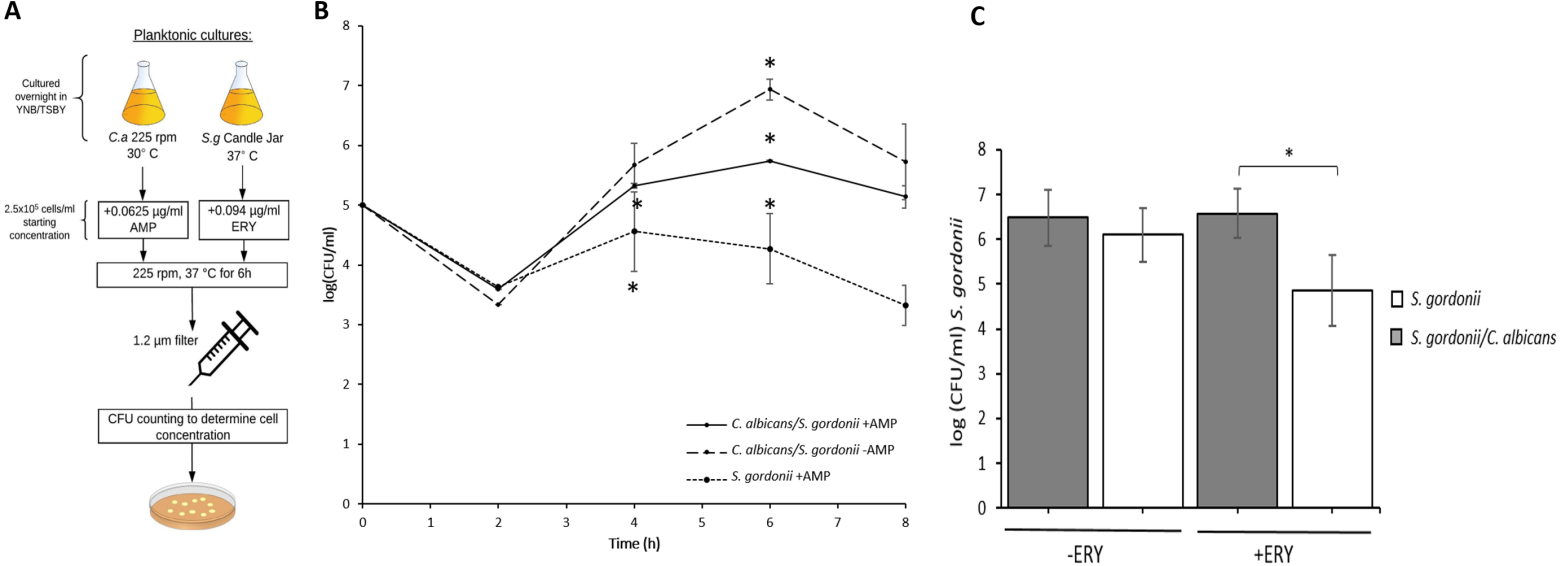
*C. albicans* provides *S. gordonii* antibiotic tolerance in planktonic co-cultures. (A) Schematic of culture conditions and antibiotic treatment. (B) CFUs (Mean+/-SD) of *S. gordonii* in mono and dual cultures with *C. albicans,* in the presence or absence of ampicillin. At 4 h, a significant difference was found between the positive control (*S. gordonii.* + ampicillin) and negative control (*C. albicans.* + *S. gordonii*, no ampicillin) and between the test group (*C. albicans* + *S. gordonii.* + ampicillin) and positive control group. No significant differences between the groups were found at 8 h. Statistical significance was observed between all groups at 6 h ( *p* < 0.05) using ANOVA with results adjusted post-hoc using Tukey’s Test. (C) CFUs (Mean ±SD) of *S. gordonii* in mono and dual cultures with *C. albicans,* with and without erythromycin at 6 h time point. There was no significant difference between *S. gordonii* survival with and without erythromycin when *C. albicans* was present. However, significantly increased killing was observed when *S. gordonii* was present alone. Three replicates were used for each experimental group and a minimum of two repeats were performed for each experiment. Statistical analysis was done using Student’s t-Test (*p* < 0.05).

Overnight cultures of wild type *C. albicans* and *S. gordonii* were inoculated from frozen stocks in YNB/TSBY (50/50) and grown in their respective preferred growth conditions overnight. The concentrations of the overnights were determined and then used to inoculate three different groups, all in a 10 ml total volume of YNB/TSBY (50/50), at a concentration of 1 × 10^5^ cells/ml. The test group was a co-culture of *C. albicans* and *S. gordonii* supplemented with ampicillin (G-Biosciences, MO) at a concentration of 0.125 µg/ml. The positive control group was a single culture of *S. gordonii* supplemented with ampicillin. The negative control group was a co-culture of *C. albicans* and *S. gordonii* with no antibiotic added. The ampicillin concentration used was based on a MIC value range found in literature (Etienne, Gruer & Fleurette, 1984; Nemoto et al., 2013). All cultures were grown at 37 °C with shaking. Samples were taken at 2, 4, 6, and 8 h and filtered through a 1.2 µm syringe filter (Whatman, GE Healthcare Life Sciences, IL) to remove *C. albicans.* The filtrate was diluted and plated on TSBY agar plates in triplicate. Plates were incubated overnight at 37 °C in a candle jar. Colony forming units (CFU) were counted the next day to determine CFU/ml. Raw data was statistically analyzed utilizing ANOVA performed using the default stats package in R Core Team v3.1.2 with results adjusted post-hoc with Tukey’s test. Please see Fig. 2A for a flow chart of methods.

### Assessment of erythromycin tolerance in planktonic cultures

Overnight cultures of wild type *C. albicans* and *S. gordonii* were inoculated from frozen stocks in YNB/TSBY and grown in their respective preferred growth conditions overnight. The concentrations of the overnights were determined and used to inoculate six different cultures in a total volume of 10 ml YNB/TSBY. Four cultures served as controls. The first two cultures were grown without antibiotic and include a single culture of *S. gordonii* and a co-culture of *C. albicans* and *S. gordonii.* The second pair of cultures, single culture of *S. gordonii* and a co-culture, were supplemented with ampicillin (0.0625 µg/ml). The last pair of cultures, single culture of *S. gordonii* and a co-culture, were supplemented with erythromycin (0.094µg/ml) (Bancescu et al., 2004). All six cultures were grown at 37 °C with shaking. Samples were taken at 2, 4, 6, and 8 h and filtered through a 1.2 µm syringe filter (Whatman, GE Healthcare Life Sciences, IL) to remove *C. albicans.* The filtrate was diluted and plated on TSBY agar plates in triplicate. Plates were incubated overnight at 37 °C in a candle jar. Colony forming units (CFU) were counted the next day to determine CFU/ml. Raw data was statistically analyzed using Student’s *t*-test. Please see Fig. 2A for a flow chart of methods.

### Analysis of dual species biofilm formation of *C. albicans* with *S. gordonii*

**Figure 3:**
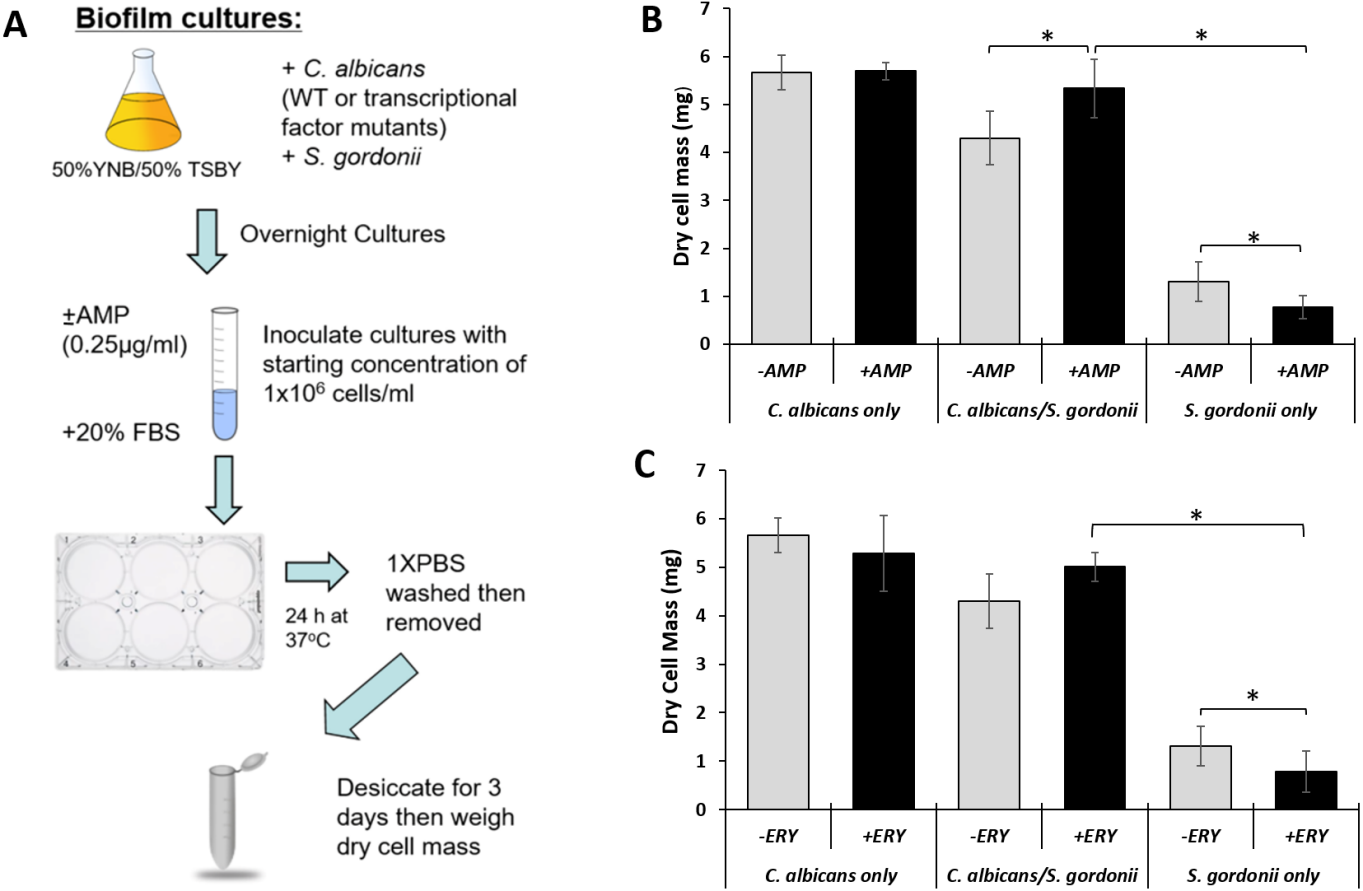
*C. albicans* provides *S. gordonii* antibiotic tolerance in biofilm co-cultures. (A) Schematic of biofilm culture conditions, antibiotic treatment and measurement of biofilm weight following dessication. (B) Dry weight measurement (Mean ± SD) of *S. gordonii* and *C. albicans* biofilms with and without ampicillin at 24 h time point. There was no significant difference between dry weights of biofilms with and without ampicillin when *C. albicans* was present. However, significantly reduced biofilm dry weight was observed for single species *S. gordonii* biofilms with ampicillin. Statistical analysis was done by Student’s t-Test (*p* < 0.05). (C) Dry weight measurement (Mean ± SD) of *S. gordonii* and *C. albicans* biofilms with and without erythromycin at 24 h time point. There was no significant difference between dry weights of biofilms with and without erythromycin when *C. albicans* was present. However, significantly reduced biofilm dry weight was observed for single species *S. gordonii* biofilms with erythromycin. Three replicates were used for each experimental group and a minimum of two repeats were performed for each experiment. Statistical analysis was done using Student’s t-Test (*p* < 0.05).

Analysis of dual species biofilms was done as described previously (Mancuso et al., 2018). Briefly, overnight cultures of wild type *C. albicans* and *S. gordonii* strains were inoculated from frozen stocks in YNB/TSBY and grown in their respective growth conditions overnight. The concentrations of the overnights were determined and used to inoculate several different cultures with the starting concentration of 1 × 10^6^ cells/ml for each organism in a 6 ml total volume of YNB/TSBY supplemented with 20% FBS. The first two cultures were grown without antibiotic and include a single culture of *S. gordonii* and a co-culture of *C. albicans* and *S. gordonii.* The second pair of cultures, single culture of *S. gordonii* and a co-culture, were supplemented with ampicillin (0.25 µg/ml). The last pair of cultures, single culture of *S. gordonii* and a co-culture, were supplemented with erythromycin (0.376 µg/ml). Each culture was transferred to uncoated 6-well polystyrene culture plates (2 ml/well) and incubated statically for 24 h at 37 °C. After incubation, the media was carefully removed and the biofilms were washed once with 1x PBS. The biofilms were removed with additional 1x PBS and transferred to pre-weighed microfuge tubes. The samples were centrifuged to pellet the cells and remove most of the liquid to facilitate drying. The sample tubes were opened and placed in a desiccator jar with anhydrous calcium chloride used as the desiccant. Dry cell mass was determined after 3 days using an analytical weighing scale. Raw data was statistically analyzed using Student’s *t*-test. Please see Fig. 3A for a flow chart of methods.

### Dual species biofilm formation and ampicillin tolerance

Biofilms were prepared as described previously (Mancuso et al., 2018). Overnight cultures of wild type and transcription factor mutant *C. albicans* strains and the wild type Challis CH1 *S. gordonii* strain were inoculated from frozen stocks in YNB/TSBY and grown in their respective growth conditions overnight. The concentrations of the overnight cultures were determined and then used to inoculate several different cultures with the starting concentration of 1 × 10^6^ cells/ml for each organism in a total volume of 6 ml of YNB/TSBY supplemented with 20% Fetal Bovine Serum (FBS) (Seradigm, VWR, GA). There were three groups and two cultures in each group. One culture for each group was grown with ampicillin (0.25 µg/ml) and the other was grown without ampicillin. The first group was a co-culture of wild type *C. albicans* and *S. gordonii*, the second was a co-culture of one of the 45 *C. albicans* transcriptional regulator mutants from the Homann knockout set and *S. gordonii*, and the third was a single culture of *S. gordonii*. Each culture was transferred to uncoated 6-well polystyrene culture plates (2 ml/well) and incubated statically for 24 h at 37 °C. After incubation, the media was carefully removed and the biofilms were washed once with 1 ×PBS. The biofilms were removed with additional 1x PBS and transferred to pre-weighed microfuge tubes. The samples were centrifuged to pellet the cells and remove most of the liquid to facilitate drying. The sample tubes were opened and placed in a desiccator jar with anhydrous calcium chloride used as the desiccant. Dry cell mass was determined after 3 days. Please see Fig. 3A for a flow chart of methods.

### Scanning electron microscopy (SEM) of biofilms

Biofilms were prepared as described previously (Mancuso et al., 2018). Briefly, overnight cultures of the wild type *C. albicans* and *S. gordonii* strains were inoculated from frozen stocks in YNB/TSBY (50/50) and grown in their respective growth conditions overnight. The concentrations of the overnight cultures were determined and then used to inoculate several different cultures with the starting concentration of 1 × 10^6^ cells/ml for each organism in a total volume of 4 ml of YNB/TSBY (50/50) supplemented with 20% FBS. Two, dual-species biofilm cultures with both organisms were set up. Mono-species biofilm cultures for *S. gordonii* and *C. albicans*, were also set up. 2 ml/well of each culture was transferred to uncoated 6-well polystyrene culture plates (Falcon, Corning, NY) containing FBS-coated glass microscope slide (Globe Scientific, NJ) pieces for the biofilms to grow on for use in SEM imaging. The culture plates were incubated statically for 24 h at 37 °C. After incubation, the media was carefully removed from the biofilm cultures. The biofilms were fixed with cold 2.5% glutaraldehyde (Electron Microscopy Sciences, PA) in 1×PBS at 4 °C for 20 min. 1×PBS was added to wash the biofilms and left on for 10 min. The biofilms were dehydrated with a series of ethanol washes lasting 5 min each including 30, 50, 70, and 90% ethanol v/v in water with two final washes of 100% ethanol. Biofilms were not allowed to dry out until the final drying step that included incubating in the chemical drying agent, hexamethyldisilazane (HMDS) (Acros Organics, Fisher Scientific, ON), for 5 min and then removal. The biofilms were then allowed to air dry completely. The biofilm samples were coated with evaporated carbon at high vacuum (Denton 502 Evaporator). SEM images were acquired with a Hitachi SU70 FESEM at 2.0 KeV using the lower detector and a 45° tilt.

## Results

### *C. albicans* and *S. gordonii* secreted proteomes in planktonic co-cultures

In order to culture *C. albicans* and *S. gordonii* in mixed planktonic and biofilm cultures, the independent growth of *C. albicans* in several combinations of yeast nitrogen base (YNB) and tryptic soy broth yeast extract (TSBY) was tested. A culture medium with YNB/TSBY (50/50) was found to be the most conducive for growth of *C. albicans* with optimal cell concentrations (Fig. S1). Additionally, the growth of *C. albicans* under various pH conditions was examined and pH 7 was found to be optimal (Fig. S2). *S. gordonii* is normally grown under static conditions. However, the co-cultures were to be cultured with shaking for *C. albicans*. Hence, we tested whether there was any difference in the cultures for *S. gordonii* in YNB/TSBY (50/50) for static and shaking conditions and found no significant differences (Fig. S3). For proteomic analysis of secreted proteins co-cultures of *C. albicans* and *S. gordonii* were prepared as depicted in the schematic in Fig. 1A. The isolated secreted proteins from the illustrates the On the silver-stained SDS-PAGE gel (Fig. 1B), the lanes for the medium alone show that proteins with a molecular weight of 22 kDa and smaller are most likely proteins from the TSBY medium. No proteins from the YNB medium formed obvious identifiable bands as it is synthetic media. *S. gordonii,* regardless of which medium it grew in, exhibited more secreted protein compared to *C. albicans.* Some proteins, like the 50-kDa protein for *S. gordonii*, were present in higher quantity in the 100% TSBY medium versus the YNB/TSBY (50/50) medium. There appeared to be more proteins released in co-culture (lane 7) and this is simply due to cumulative secretion of proteins by both microorganisms. However, it is unclear which proteins belong to which organism on the gel. The majority of these proteins may possibly belong to *S. gordonii* since it produced more proteins in YNB/TSBY (50/50) medium than *C. albicans* did in the same medium. All lanes were equally loaded with the exception of YNB alone since it is a synthetic media (lane 1) and the *C. albicans* single culture grown in YNB since it secretes very little protein when grown in this media (lane 5).

The first nano-LC/MS/MS analysis identified 85 proteins in the *C. albicans* single culture (all samples were obtained YNB/TSBY (50/50)). A total of 237 proteins were identified in the *S. gordonii* single culture. A total of 348 proteins were identified in the co-culture, with 80 of those belonging to *C. albicans* and 268 belonging to *S. gordonii.* The second MS analysis identified 208 proteins in the *C. albicans* single culture. A total of 468 proteins were identified in the *S. gordonii* single culture. A total of 539 proteins were identified in the co-culture, with 137 of those belonging to *C. albicans* and 402 belonging to *S. gordonii*. The MS analyses used three genomic databases to rule out protein contamination from other sources (such as the media) and to verify that the proteins did belong to either *C. albicans* or *S. gordonii*. The results from the first and second MS analyses were combined. Proteins present in both the first and second analyses for *C. albicans* in single culture were identified, as they were for *S. gordonii* in single culture. Proteins present in both the first and second analyses for *C. albicans* and *S. gordonii* in co-culture were also identified. Thirteen proteins from *C. albicans* were identified in the single culture. One protein was identified for *S. gordonii* in single culture. In co-culture, four proteins belonged to *C. albicans* and one protein belonged to *S. gordonii*, for a total of 5 proteins identified in co-culture (Table 1).

**Table 1:** Secreted proteome of *C. albicans* and *S. gordonii* interactions in planktonic cultures. Proteomic analysis was performed by nano-LC/MS/MS analysis. Proteins identified in two separate analyses have been listed in the table.

	*Candida albicans*	*Streptococcus gordonii*
Single culture only proteins	Translation initiation factor	Conserved hypothetical protein TIGR00096
	Cytosolic ribosomal protein L12	
	Elongation factor 2	
	Elongation factor 3	
	Adenosine kinase	
	Hypothetical protein CaO19.10988	
	Likely cytosolic ribosomal protein L3	
	Galactose/glucose transporter	
	Hypothetical protein CaO19.6673	
	Sphingolipid long chain base-responsive protein LSP1	
	Ribosomal protein 10	
Co-culture only proteins	Hypothetical protein 76573724	Ethanol-active dehydrogenase/acetaldehyde-active reductase
	Hypothetical protein CAWG_05665	
	SNF-2 family ATP dependent chromatin remodeling factor snf21	
	Conserved hypothetical protein 238879991	

### Antibiotic tolerance in planktonic co-cultures and dual species biofilms

In order to assess the role of *C. albicans* in resistance to ampicillin in planktonic co-cultures with *S. gordonii*, they were cultured in YNB/TSBY (50/50) with ampicillin (0.25 µg/ml) and CFU/ml were analyzed every 2 h for 8 h using a protocol described in Fig. 2A. As seen in Fig. 2B, *S. gordonii* growth for all groups deceased for all groups at 2 h, then increased at 4 and 6 h before finally decreasing at 8 h. At 4 h, a significant difference was found between the test (*C. albicans* + *S. gordonii* + ampicillin) and negative control (*C. albicans* +*S. gordonii* - ampicillin) groups. At 6 h, all groups were significantly different, and at 8 h no significant differences were found between any of the groups. To test whether tolerance of co-cultures extends to other antibacterial agents, the cultures were incubated with erythromycin for 6 h and *S. gordonii* CFUs were analyzed. The data show that in co-cultures *S. gordonii* CFUs were significantly higher indicating protection from erythromycin (Fig. 2C). This finding suggests that *C. albicans* has a protective effect on *S. gordonii* when it is grown in the presence of antibacterial agents. This tolerance to antibiotics was also observed for dual species biofilms of *C. albicans* and *S. gordonii* (Fig. 3)*.* For the dual species biofilms with antibiotics, biofilm formation was more than the no antibiotic group (Fig. 3B & Fig. 3C). In fact, for the ampicillin group there was a significantly increased biofilm formation for dual species. There is a possibility that this increase in biofilm formation by weight in the antibiotic group could be because of an increase in proliferation of *C. albicans* in the biofilm while *S. gordonii* struggles to grow under antibiotic stress. This may be further supported by the finding that mono-species biofilm formation is at similar levels to the antibiotic treated dual species biofilms. These data indicate that dual species biofilms may be more tolerant to ampicillin and erythromycin.

### Binding and coaggregation of *C. albicans* and *S. gordonii*

**Figure 4:**
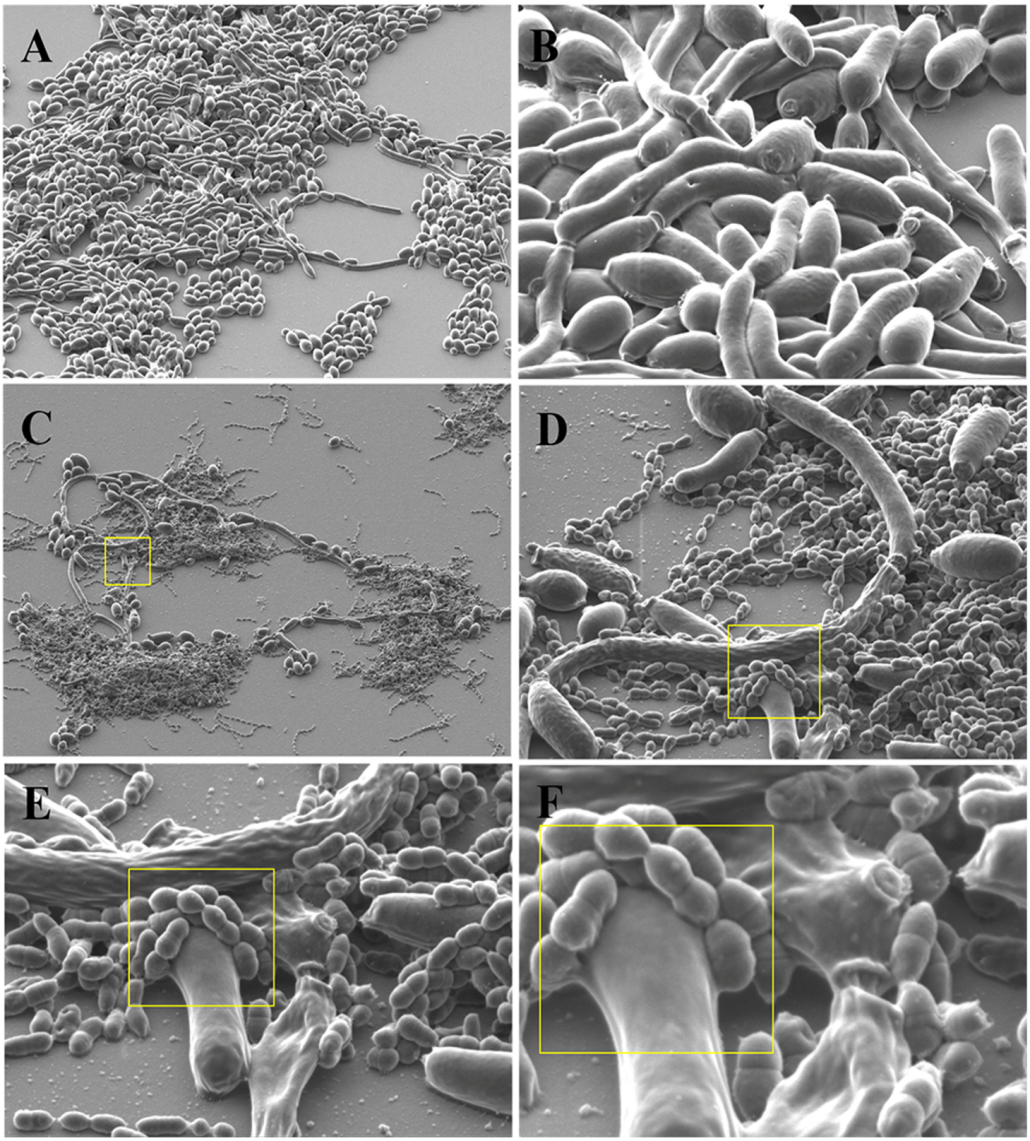
Binding and coaggregation of *S. gordonii* and *C. albicans* in early biofilms. Scanning Electron Microscopy (SEM)****images were acquired with a Hitachi SU70 FESEM at 2.0 KeV using the lower detector and a 45° tilt. (A) *C. albicans* biofilm at 1,000× magnification. (B) *C. albicans* biofilm at 5,000× magnification. (C) *C. albicans* and *S. gordonii* dual species biofilm at 1,000× magnification. (D) *C. albicans* and *S. gordonii* dual species biofilm at 5,000× magnification. (E) *C. albicans* and *S. gordonii* dual species biofilm at 10,000× magnification. (F) *C. albicans* and *S. gordonii* dual species biofilm at 20,000× magnification.

SEM analysis of early biofilms cultured in YNB/TSBY (50/50) with 10% fetal bovine serum (FBS) revealed coaggregation of *C. albicans* and *S. gordonii* (Fig. 4). *S. gordonii* cells were found to be growing comfortably on *C. albicans* yeast and hyphae and appeared to be firmly attached to the candida cell wall (Figs. 4C–4D).

### *C. albicans* cell wall integrity transcription factors affect dual species biofilm formation with *S. gordonii* as well as antibacterial tolerance

**Figure 5:**
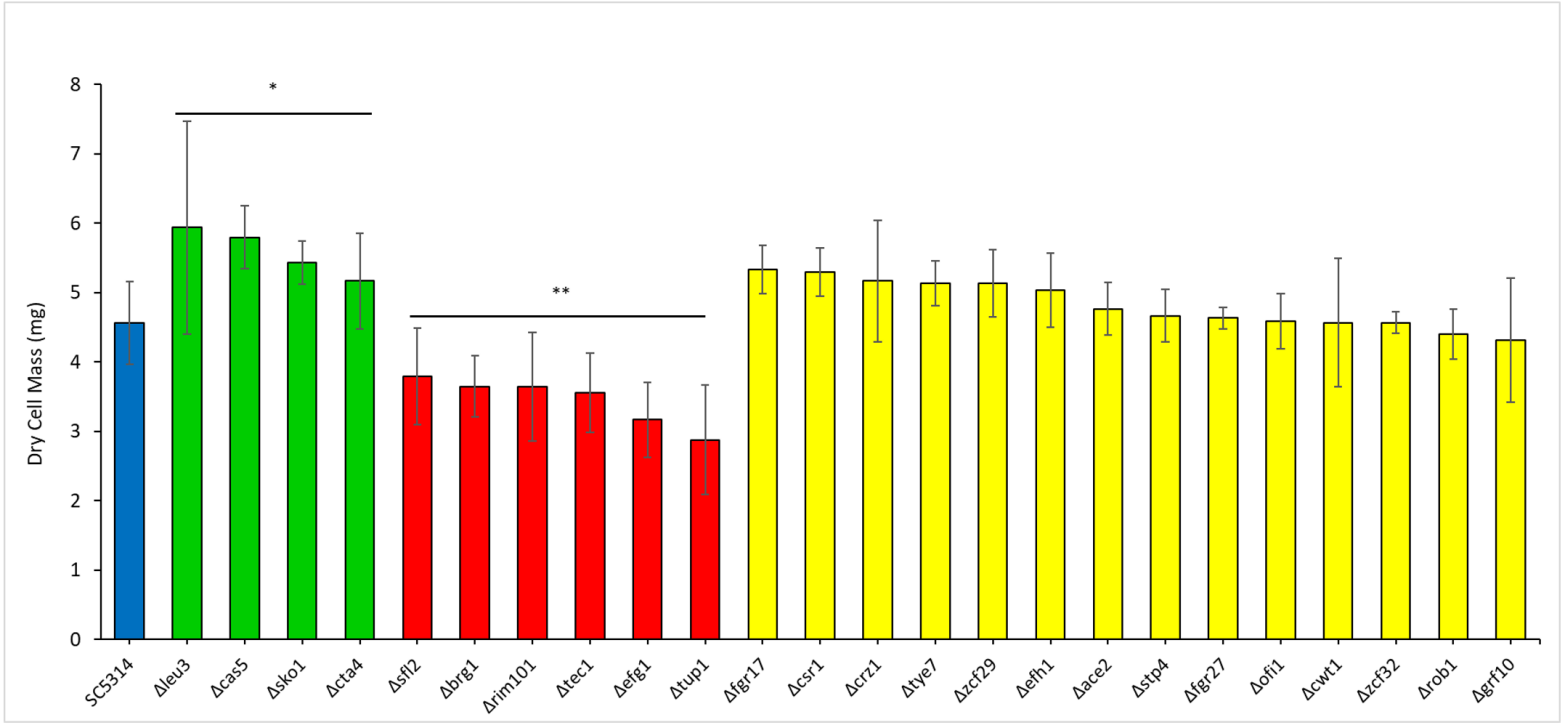
Transcriptional regulators affect the ability of *C. albicans* to form polymicrobial biofilms with *S. gordonii*. Dry weights were measured for biofilms of *C. albicans* transcription factor Knock-out mutants with *S. gordonii* and compared with biofilms of WT *C. albicans* and *S. gordonii*. Statistical analysis was done using Student’s t-Test (*p* < 0.05).

In order to assess polymicrobial biofilm formation *C. albicans* cell wall integrity transcription factor mutants (Fig. 5) were cultured as dual species biofilms with *S. gordonii*. The dry weight of the resulting biofilms was compared with those formed by WT *C. albicans* with *S. gordonii*. Interestingly, mutants of transcription factors leu3, cas5, cta4 and sko1 formed significantly heavier biofilms while mutants of sfl2, brg1, tec1, tup1, efg1 and rim101 formed significantly lighter biofilms (Fig. 5). Additionally, dual species biofilms of *S, gordonii* and sfl2, tec1 and efg1 transcription factor mutants of *C. albicans* also displayed reduced tolerance to antibiotics.

## Discussion

Biofilms are composed of microbial organisms embedded in extracellular matrix. In a *C. albicans* biofilm it has been documented that 55% of the dry weight of the extracellular matrix is composed of proteins (Zarnowski, Sanchez & Andes, 2016). It has also been found that there is a striking resemblance in glycoproteins that are part of the extracellular matrix of the biofilm as well as supernatants of planktonic cultures (Thomas, Bachmann & Lopez-Ribot, 2006). Thus it is very likely that glycoproteins secreted during planktonic cultures will end up in the extracellular matrix during growth as biofilms (Pierce et al., 2017). Moreover, in polymicrobial biofilms, proteins secreted by microorganisms may play important roles in interactions. Hence our proteomic analysis of secreted proteins in dual planktonic cultures of *C. albicans* and *S. gordonii* is justified and may represent the proteins being secreted in their biofilms as well. For determining optimal culture conditions for dual cultures, we tested the growth of *C. albicans* in mixed media at various concentrations and found that TSBY/YNB (50/50) was the most optimal for growth (Fig. S1). The pH of our various media mixtures ranged from 5.4 to 7.02, acidic to neutral, however, this did not seem to significantly impact the growth of *C. albicans* (Fig. S2). Since we planned to study mixed cultures of *C. albicans* with *S. gordonii*, which prefers TSBY medium, we chose TSBY/YNB (50/50) medium without pH adjustment as our co-culture media. *S. gordonii* is usually cultured in static conditions in the laboratory. However, the dual planktonic cultures were to be under shaking conditions for optimal growth of *C. albicans*. Hence, we compared growth of *S. gordonii* in TSBY/YNB (50/50) medium at static and shaking conditions and found no significant differences (Fig. S3). Although *S. gordonii* appeared to produce more protein bands in both single and co-cultures compared to *C. albicans,* when samples were analyzed using nano-LC/MS/MS, more differentially secreted protein bands were identified for *C. albicans* (Table 1)*.* However, *C. albicans* may have produced more protein bands if the co-cultures had been allowed to incubate for more than the allotted 30 min, which only minimally offers eukaryotes time to translate protein. The proteomic analysis data presented in this manuscript is purely qualitative and the protein bands may not represent individual proteins but may also be processed proteins and thus future quantitative Mass Spectrometry analysis will be needed to ascertain the quantity and identity of those protein bands. Furthermore, limitations of proteomic analysis in this study include the limited number of repetitions (*n* = 2), and the variable environments used for culture conditions. Many of the proteins that were differentially secreted appeared to be non-classical, i.e., without a signal peptide. This has been observed in past studies of proteomic analysis as well (Chaffin et al., 1998; Maddi, Bowman & Free, 2009; Nombela, Gil & Chaffin, 2006). One of these differentially secreted proteins non-classical protein, SNF2-family ATP dependent chromatin remodeling factor Snf21, is essential for cell viability and is needed for mitotic progression (Yamada et al., 2008). There is evidence that this complex regulates the expression of a gene that promotes adhesion for cell-to-cell contact, biofilm formation and the dimorphic switch (Barrales et al., 2012). Since this protein appears in co-culture, it would be interesting to investigate if this protein was present because genes related to adhesion with *S. gordonii* were being upregulated. Three different hypothetical proteins without identified functions unique to *C. albicans* were also found in the co-culture only. These proteins of unknown function will be a source of interest for future studies as they may have possible implications in increased biofilm formation and virulence for either, or both, *C. albicans* and *S. gordonii.* There are multiple proteins identified in the single culture only for *C. albicans.* These include translation initiation factor, likely cytosolic ribosomal protein L12, elongation factor 2 (EF-2) and 3 (EF-3), likely cytosolic ribosomal protein L3, and ribosomal protein 10. EF-3 is unique to all fungi (Ross-Smith et al., 1995). EF-2 and EF-3 are both necessary for protein synthesis. EF-2 is encoded by one single gene (Mendoza et al., 1999), therefore, the presence of both EF-2 and EF-3 requires the activation of more than one gene. The genes that encode these proteins are on different chromosomes; EF-3 is encoded by a gene on chromosome 5 (Myers, Fonzi & Sypherd, 1992). Examples of proteins that may have been located in the cell wall and been either released from the cell wall or secreted include galactose/glucose transporter and sphingolipid long chain base-responsive protein LSP1. Adenosine kinase was identified, which is a cellular protein integral to multiple cellular functions. Additional unknown proteins that were identified that may be a source of interest for future studies include four hypothetical proteins. Only one protein was identified in co-culture that was unique to *S. gordonii*: ethanol-active dehydrogenase/acetaldehyde-active reductase. The increase in this protein may indicate an increase in cellular glycolysis. Only one protein, a conserved hypothetical protein, was identified in single culture that was unique to *S. gordonii*. This protein of unknown function may be a source of interest for future studies.

Fungi and bacteria in biofilms show increased resistance to antibiotics, including ampicillin (Donlan & Costerton, 2002). Past studies show that *C. albicans* can enhance antibacterial resistance/tolerance of gram positive bacteria like *Staphylococcus aureus* (Scheres & Krom, 2016). In that study, it was shown that attachment of *S. aureus* to *C. albicans* hyphae resulted in decreased susceptibility of the bacteria to antibiotic treatment. Furthermore, it was found that co-inoculation of *S. aureus* with *C. albicans* resulted in a more widespread infection as compared to either microorganism alone (Scheres & Krom, 2016). Similarly, another study of biofilms of *S. gordonii* with *C. albicans* found that there was enhanced resistance to antibiotics for dual species biofilms as opposed to individual biofilms (Montelongo-Jauregui et al., 2016). Based on this information, we expected that the interaction between planktonic *C. albicans* and *S. gordonii* in planktonic cultures could result in antibiotic tolerance to ampicillin and erythromycin which were routinely used for treating oral infections (Etienne, Gruer & Fleurette, 1984; Nemoto et al., 2013). The MIC concentration for ampicillin for *Streptococci* has been well established (Nemoto et al., 2013). In this reference the 0.25 microgram/ml has been determined as a concentration of antibiotic susceptibility for *Streptococci* (Nemoto et al., 2013). Thus we have utilized the above concentration for ampicillin. For Erythromycin, a range of 0.16–4 microgram/mL has been provided as MIC as described previously (Bancescu et al., 2004). We initially did some quick screening experiments to determine the Mic of 0.375 microgram/mL as ideal for our experiments.

*S. gordonii* killing was more prominent when incubated with ampicillin, however protection from ampicillin was observed by significantly increased survival of *S. gordonii* when *C. albicans* was present in the culture (Fig. 2B). This protection from ampicillin was observed at the 4, 6 and 8 h time points but it was statistically significant at 6 h. Overall, our data suggest that *C. albicans* provides a survival advantage to *S. gordonii* in the presence of ampicillin. Furthermore, this antibiotic protection in plaktonic cultures was also observed with other antibiotics such as erythromycin (Fig. 2C). We further show that dual species biofilms of *C. albicans* and *S. gordonii* are more resistant to ampicillin and erythromycin (Fig. 3). This is consistent and confirmatory with other studies (Montelongo-Jauregui et al., 2016).

Coaggregation between *C. albicans* and *S. gordonii* has been documented utilizing confocal microscopy and electron microscopy as well as light microscopy (Zijnge et al., 2010). As such coaggregation typically occurs with *S. gordonii* cells bound to hyphae of *C. albicans* in a biofilm environment (Zijnge et al., 2010). Our SEM experiments of early biofilms, using our unique culture media and conditions, confirm the binding of *S. gordonii* to *C. albicans* hyphae as well as yeast form cells (Fig. 4). In our analysis, the bacterial cells appear to be bound firmly, as if fused to the cell wall of *C. albicans* (Fig. 4). The induction of hyphae has been thought to increase the surface area and protein adhesins available for increased *S. gordonii* to *C. albicans* cell-to-cell binding (Bamford et al., 2015). This binding can play a role in biofilm formation and virulence (Bamford et al., 2009). Based on this information we hypothesized that *C. albicans* cell wall integrity had a role in the dual species biofilm formation with *S. gordonii*. To test this we screened knock-out mutants of 45 *C. albicans* transcription factors (Table 2), that regulate key functions like cell to cell adherence, hyphae formation, biofilm formation and cell membrane maintenance, for dual species biofilm formation with *S. gordonii*. Among the 45 transcription factors, we found that knock-outs of four factors - Leu3, Cta4, Cas5 and Sko1 had increased biofilm formation as compared to WT dual species biofilms, indicating that these factors negatively regulated dual species biofilms (Fig. 5). On the other hand, we found that knock-outs of six factors –Sfl2, Brg1, Tec1, Tup1, Efg1 and Rim101 had reduced dual species biofilm formation as compared to WT dual species biofilms, indicating that these factors positively regulated dual species biofilms (Fig. 5). Some of these transcription factor mutants are inherently affected in biofilm formation. Hence, we also measured biofilm formation of these mutant strains in mono-species biofilms (Fig. S4). Cas5 and Cta4 mutants had significantly increased while Sfl2, Tec1 and Rim101 had significantly decreased mono-species biofilm formation indicating that even the dual species biofilm formation could be affected by their inherent abnormalities in biofilm formation (Fig. S4). However, the remaining transcription factor mutants were unaffected in dual species biofilm formation, by their inherent abnormalities in mono-species biofilm formation (Fig. S4).

**Table 2:** List of *C. albicans* transcription factor knock-out mutants that were used in this study. The mutant strains were sourced from the Hommann plates obtained from Fungal Genetics Stock Center (FGSC).

*Transcription Factors*		*Functions*
*SFL2, GRF10, BRG1, OFI1, ZCF29, EFH1, BAS1, ZCF3, CSR1, ASH1, FGR17, TUP1, RCA1*		Involved in regulation of filamentous growth
*LEU3, CAS5, ZCF31, TRY4, ACE2, MRR2, TRY6, UGA33, SUC1, ZCF39, CZF1, BCR1*		Required for yeast cell adherence
*FGR27*		Involved in yeast cell adherence and filamentous growth
*TYE7, ZCF32, UGA32, CUP9, CTA4, ZAC7, OPI1, INO4, ROB1, GZF3, TEC1, SSN6, RIM101, CPH2, EFG1*		Required for biofilm formation, involved in regulating hyphal growth
*CWT1, SKO1*		Involved in cell wall architecture
*STP4*		Induced in core caspofungin response, colony morphology-related gene regulation by SSN6
*CRZ1*		Role in maintenance of membrane integrity

### Negative regulators of dual species biofilm formation

Leu3 is a Zn(II)2Cys6 transcription factor which functions in branched chain aminoacid synthesis. Leu3 is induced by Mnl1 under weak acid stress conditions (Ramsdale et al., 2008). A Leu3 homozygous null mutant is viable (Finkel et al., 2012). Leu3 is required for positive regulation of cell adhesion in a monospecies biofilm. Additionally Leu3 is also required for cell adhesion to a silicone substrate (Finkel et al., 2012). Cta4 is a Zn(II)2Cys6 transcription factor which is induced under weak acid stress conditions (Bamford et al., 2015). It is repressed upon adherence to polystyrene (Finkel et al., 2012). Cta4 virulence is reduced in a mouse model (Chiranand et al., 2007). Cas5 is a zinc finger transcription factor that plays a critical role in cell wall integrity response. It is required for the induction of caspofungin responsive genes in *C. albicans* including *ECM33* and *CRH11* (Chiranand et al., 2007). Cas5 is also required for virulence in mouse and drosophila infection models (Chamilos et al., 2009). Sko1 is a bZip transcription factor that is involved in cell wall damage response (Rauceo et al., 2008). It is known to repress yeast to hyphal transformation and regulate oxidative stress response in *C. albicans* (Alonso-Monge et al., 2010). Regulation of oxidative and osmotic stress response by Sko1 occurs in a Hog1 dependent manner (Enjalbert et al., 2006).

### Positive regulators of dual species biofilm formation

Sfl2 is a transcription factor involved in regulation of morphogenesis. Null mutant of Sfl2 has decreased hyphal growth and is required for infection in a reconstituted epithelial infection model (Spiering et al., 2009). Additionally, Sfl2 regulates transcriptional response dependent on sensing carbon dioxide levels (Tao et al., 2017). Brg1 is a transcription factor that plays a role in transcriptional response of hypha specific genes. Hence the null mutant has a decreased hyphal formation and virulence in mice (Cleary et al., 2012). Additionally, Brg1 null mutants have decreased biofilm formation in spider medium (Lin et al., 2013). The reduced biofilms of Brg1 mutants is observed for both conventional and pheromone-induced biofilms indicating its important role in cell cohesion (Lin et al., 2013). Tec1 is a TEA/ATTS transcription factor that is predominantly expressed in the hyphae. Tec1 deletion leads to reduced hypha formation *in vitro*, but normal hypha formation *in vivo* (Schweizer et al., 2000). Tec1 has been shown to regulate Bcr1 which is required for biofilm formation. Bcr1 induces several cell surface proteins and adhesins that are expressed during hyphal development (Nobile & Mitchell, 2005). Tup1 is a transcriptional repressor of filamentous growth. Tup1 null mutant grows only as filaments under various conditions. Tup1 null mutants produce 17-fold increased farnesol in biofilms (Nett Jeniel et al., 2009). Tup1 represses hypha specific genes and cell wall adhesins and is regulated by Tor1 (Bastidas, Heitman & Cardenas, 2009). Efg1 is a basic-helix-loop-helix (bHLH) transcription factor. It is a major transcriptional regulator that is involved in hyphal morphogenesis (Stoldt, 1997). Efg1 has a central role as a downstream component of protein kinase A (PKA) in the regulation of yeast to hypha transition (Bockmühl & Ernst, 2001). Rim101 is a zinc finger transcription factor that is involved in regulation of yeast to hypha transition in response to pH changes (Davis et al., 2000). Specifically, Rim101 induces alkaline responsive genes while repressing acid responsive genes (Baek, Martin & Davis, 2006). Additionally, null mutants of Rim101 are defective in virulence in a mouse model of disseminated candidiasis (Davis et al., 2000).

**Figure 6:**
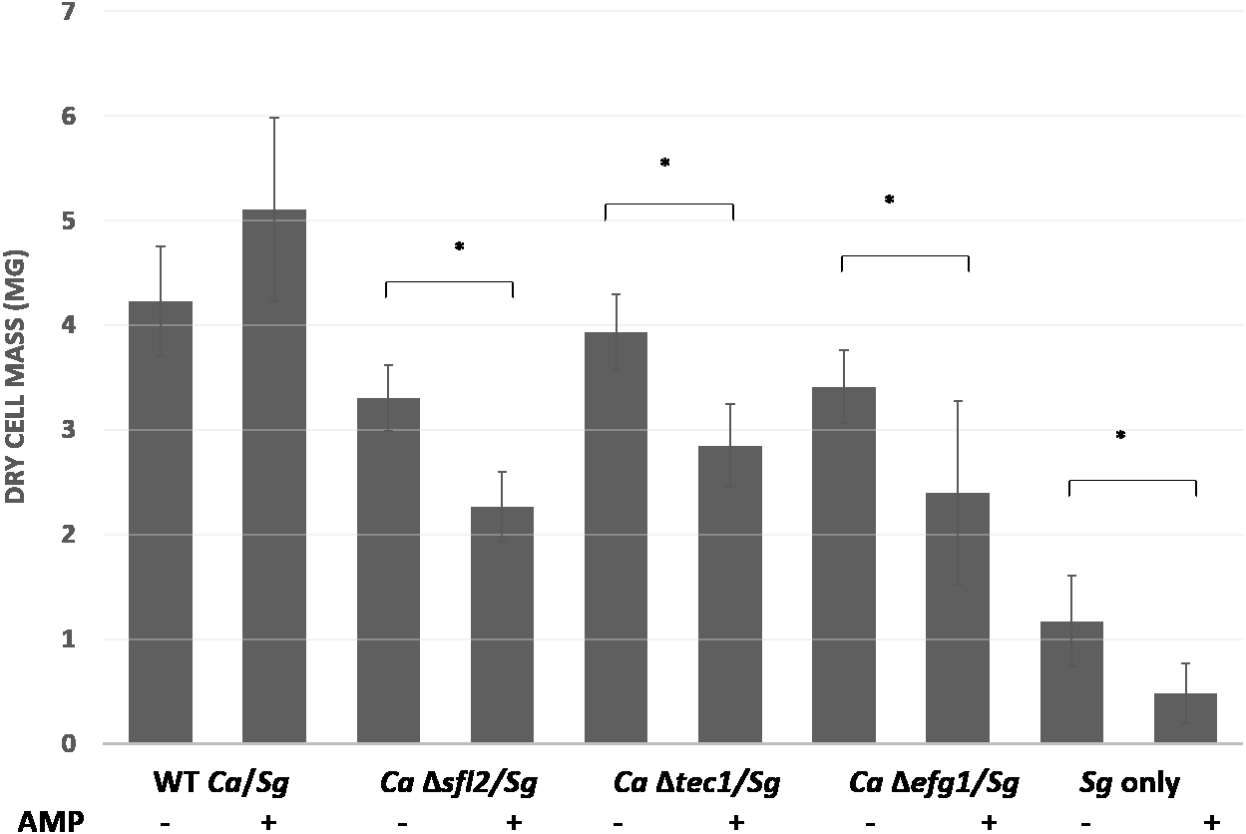
*C. albicans* transcriptional regulators Sfl2, Tec1 and Efg1 affect ampicillin resistance in polymicrobial biofilms with *S. gordonii*. Dry weights were measured for biofilms of *C. albicans* transcription factor Knock-out mutants with *S. gordonii* and compared with biofilms of WT *C. albicans* and *S. gordonii* in the presence or absence of ampicillin. Statistical analysis was done using Student’s t-Test (*p* < 0.05).

**Figure 7:**
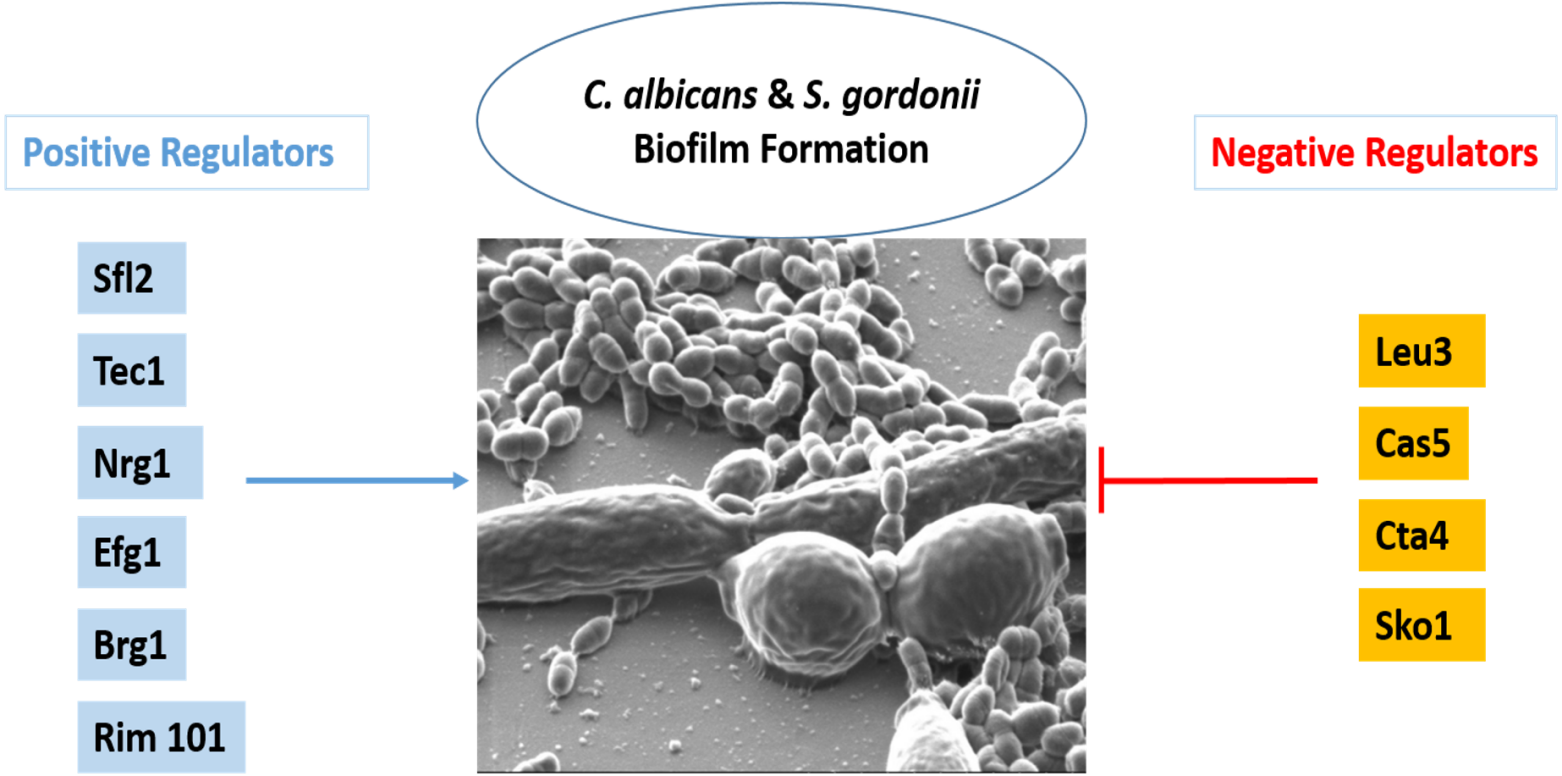
Transcriptional regulation of *C. albicans* and *S. gordonii* biofilms. Please see Table 2 for functions related to the transcription factors in this figure.

The *C. albicans* transcription factors which may negatively regulate dual species biofilm formation with *S. gordonii* are mainly involved in candida cell–cell adhesion, adhesion to a surface or cell wall integrity. It is reasonable that repression of candida cell to cell adhesion may be needed in order to promote cell adhesion with *S. gordonii* for a mixed biofilm formation. Thus the functions of transcription factors especially Leu3 and Cta4 might be important for adherence of *C. albicans* with *S. gordonii*. Cell wall integrity or cell wall damage response usually occurs in response to stress and is mediated by the Cek1 pathway in *C. albicans* (Román et al., 2015). Cell wall integrity is very important for cell survival and hence several transcription factors regulate this key function. Thus it is possible that although Cas5 and Sko1 play a role in cell wall integrity and damage response they might not be required for the biofilm formation with *S. gordonii* due to compensatory effect by other transcription factors. On the other hand, the transcription factors Sfl2, Brg1, Tec1, Tup1, Efg1 and Rim101 are mainly required for yeast to hyphal morphogenesis and thus also play critical roles in candida biofilm formation. A reduced biofilm formation of mutants of these transcription factors with *S. gordonii* indicates that hypha formation by *C. albicans* is critical for robust biofilm formation. Furthermore, we also screened the knock-out mutants of all 45 transcription factors in order to identify putative regulators of ampicillin resistance in dual species biofilms with *S. gordonii*. We observed that knock-out mutants of Sfl2, Efg1 and Tec1 demonstrated significantly decreased biofilm formation with *S. gordonii* in the presence of ampicillin as compared to control biofilms indicating a reduced antibiotic resistance (Fig. 6). This data indicates a role for these transcription factors in antibiotic resistance of dual species biofilms (Fig. 7).

## Conclusions

Differential protein secretion occurs in dual species planktonic cultures of *C. albicans* and *S. gordonii* indicating a possible way of interaction between the two microorganisms. Additionally, antibiotic resistance to ampicillin and erythromycin was observed in both planktonic and biofilm cultures of *C. albicans* and *S. gordonii.* Binding between these oral microorganisms in early biofilms was confirmed using SEM analysis. Additionally, *C. albicans* transcription factors play a key role in dual species biofilm formation and ampicillin resistance of *S. gordonii.* This study can serve as a template for other studies investigating the relationship of *C. albicans* with bacteria in polymicrobial biofilms.

##  Supplemental Information

10.7717/peerj.7870/supp-1 Figure S1 *C. albicans* growth curves under various media conditions

10.7717/peerj.7870/supp-2 Figure S2 *C. albicans* growth curves under various pH conditions with YNB/TSBY (50/50)

10.7717/peerj.7870/supp-3 Figure S3 *S. gordonii* growth curves under shaking versus static conditions

10.7717/peerj.7870/supp-4 Figure S4 Mono-species biofilm formation by *C. albicans* transcription factor mutants Biofilms were cultured for 24h in YNB with serum as described in methods. Biofilms were dried and weighed. WT (SC5314) strain was used as control.

## Additional Information and Declarations

### Data Availability

The following information was supplied regarding data availability: Maddi, Abhiram (2019): Raw Data for PeerJ manuscript 38361. figshare. Dataset. https://doi.org/10.6084/m9.figshare.9862856.v1.
