# Supplementary figures and images for: Candida albicans cell wall integrity transcription factors regulate polymicrobial biofilm formation with Streptococcus gordonii
Source: PeerJ. 2019 Oct 11;7:e7870. doi: 10.7717/peerj.7870 (PMC6791342; doi:10.7717/peerj.7870)

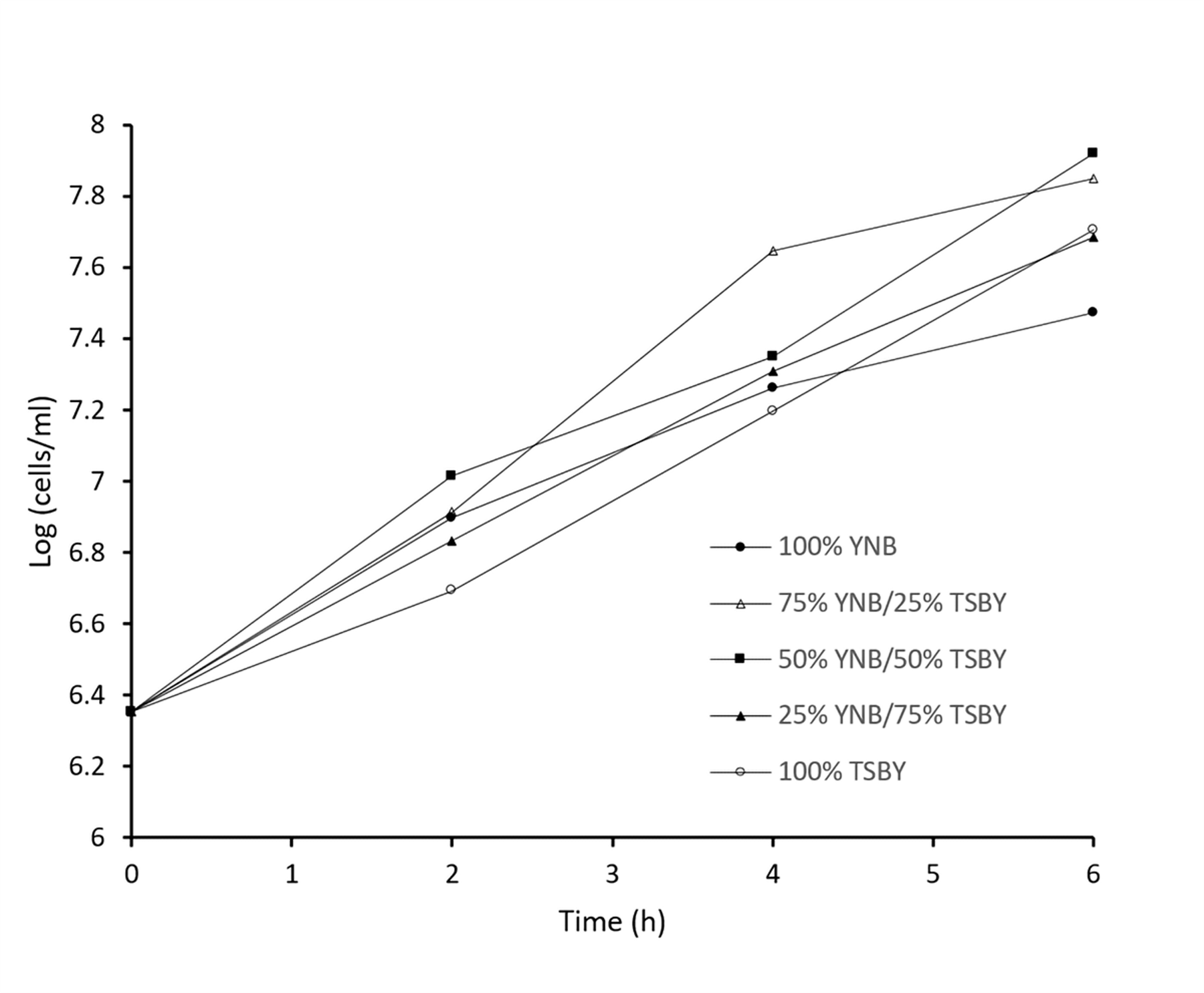

Supplement: Figure S1 [file peerj-07-7870-s001.png]

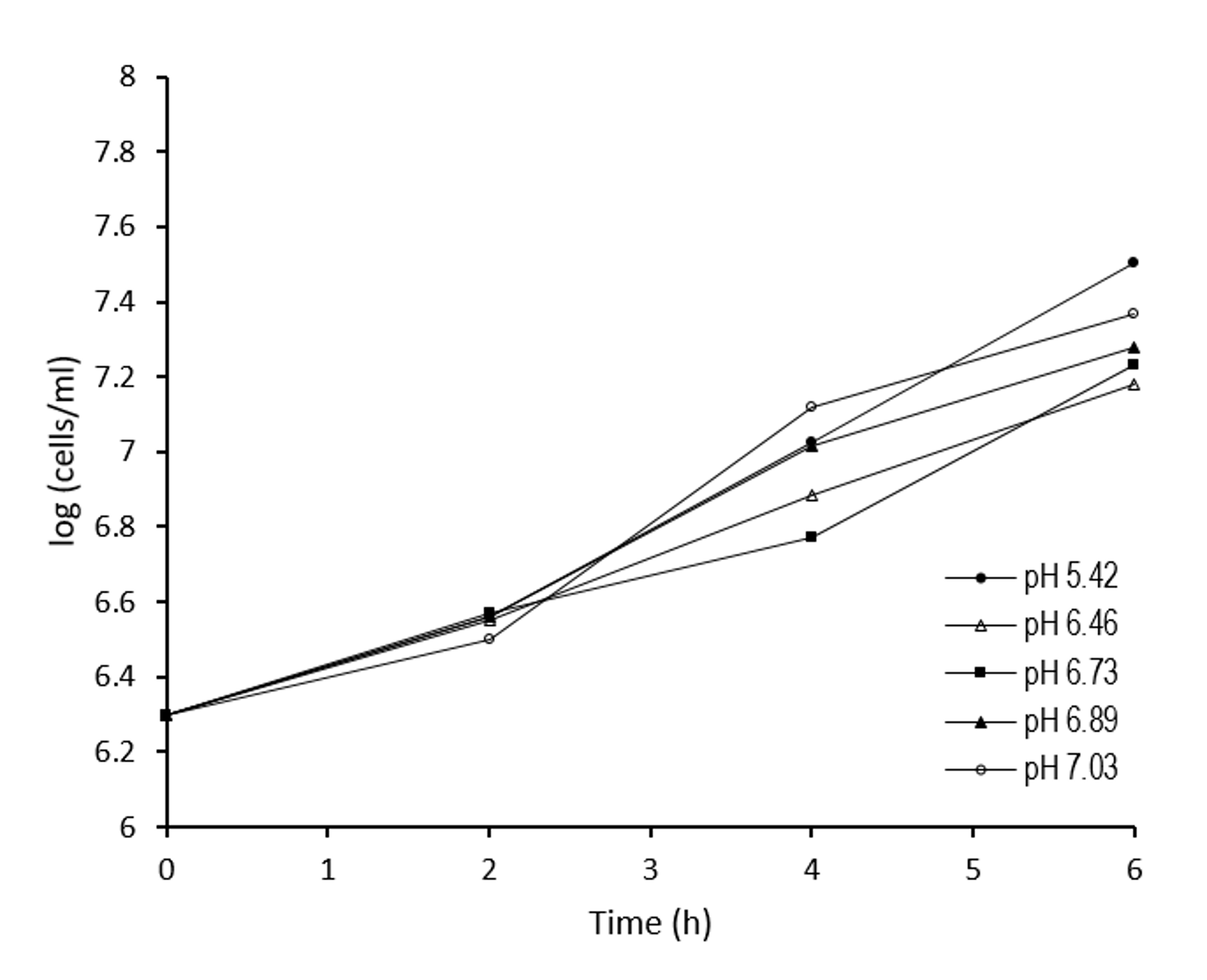

Supplement: Figure S2 [file peerj-07-7870-s002.png]

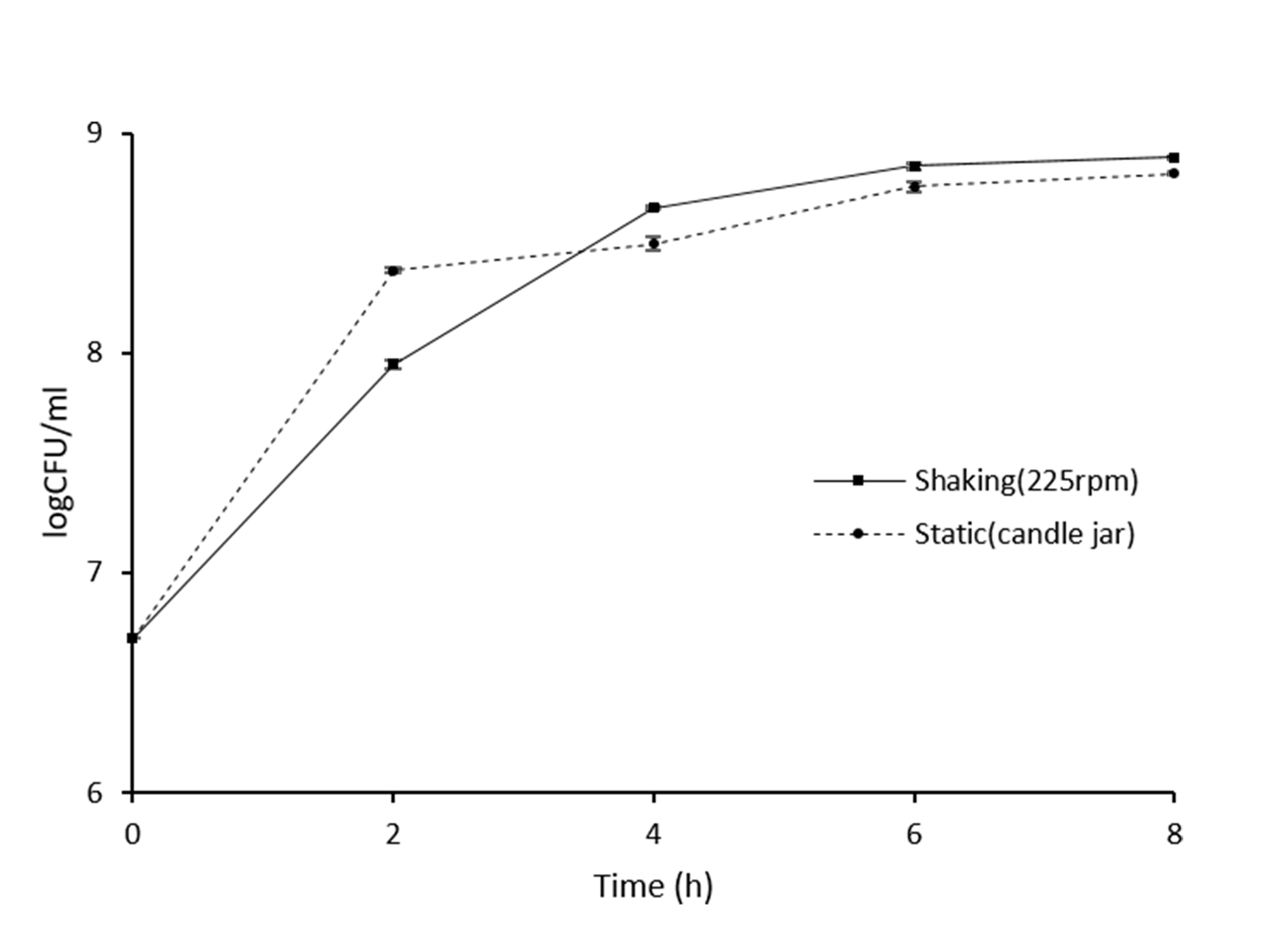

Supplement: Figure S3 [file peerj-07-7870-s003.png]

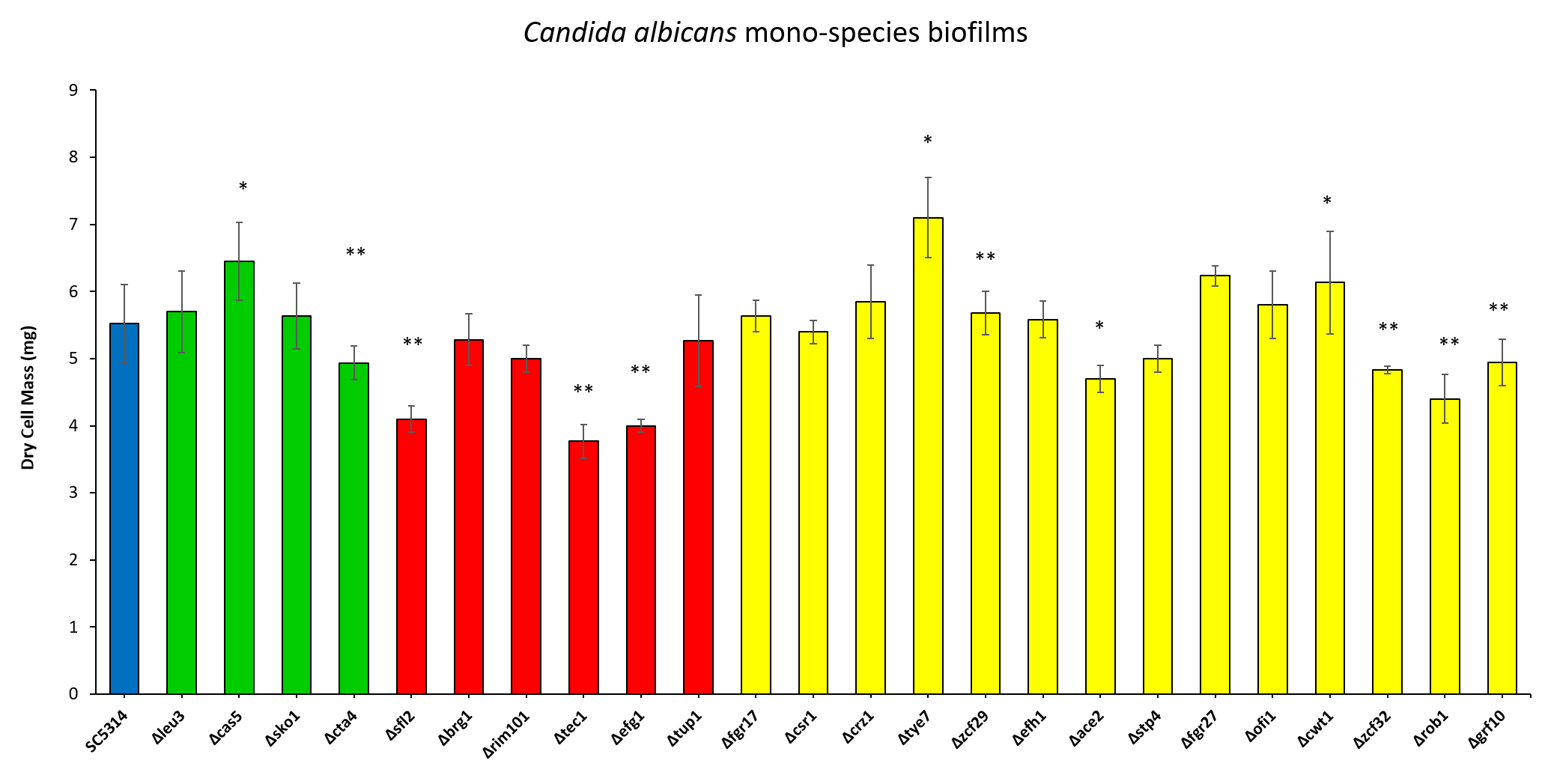

Supplement: Figure S4 — Biofilms were cultured for 24h in YNB with serum as described in methods. Biofilms were dried and weighed. WT (SC5314) strain was used as control. [file peerj-07-7870-s004.png]
